# Gut microbiota involved in desulfation of sulfated progesterone metabolites: A potential regulation pathway of maternal bile acid homeostasis during pregnancy

**DOI:** 10.3389/fmicb.2022.1023623

**Published:** 2022-10-20

**Authors:** Peng Wang, Qianqian Chen, Peiqiang Yuan, Sen Lin, Hong Chen, Ran Li, Xiaoling Zhang, Yong Zhuo, Jian Li, Lianqiang Che, Bin Feng, Yan Lin, Shengyu Xu, De Wu, Zhengfeng Fang

**Affiliations:** ^1^Key Laboratory for Animal Disease Resistance Nutrition of the Ministry of Education, Animal Nutrition Institute, Sichuan Agricultural University, Chengdu, China; ^2^College of Biological Engineering, Henan University of Technology, Zhengzhou, China; ^3^Sericultural and Agri-Food Research Institute, Guangdong Academy of Agricultural Sciences, Guangzhou, China; ^4^Key Laboratory for Food Science and Human Health, College of Food Science, Sichuan Agricultural University, Ya’an, China

**Keywords:** bile acid homeostasis, pregnant sows, gut microbiota, vancomycin, sulfated progesterone metabolites

## Abstract

Abnormally raised circulating bile acids (BA) during pregnancy threat fetal and offspring health. Our previous study has identified sulfated progesterone metabolites (PMSs) in part account for dysregulation of maternal BA homeostasis during pregnancy, however, limited intervention strategies to remedy increased serum BA through PMSs during pregnancy are available. The purpose of this study is to test the feasibility of manipulating BA homeostasis and progesterone metabolism through steering gut microbiota. A total of 19 pregnant sows were randomly treated with standard diet or vancomycin-supplemented diet, to investigate the intercorrelation of PMSs, intestinal microbiota, and maternal BA metabolism from day 60 of gestation (G60) until farrowing (L0). Pregnant mice orally gavaged with epiallopregnanolone sulfate (PM5S) or vehicle and nonpregnant mice were sampled and further analyzed to verify the effect of PM5S on maternal BA metabolism. The present study revealed that oral vancomycin reduced maternal fasting serum total BA (TBA) levels and postprandial serum TBA levels at day 90 of gestation (G90). BA profile analysis showed the decreased TBA after vancomycin treatment was attributed to the decrease of primary BA and secondary BA, especially hyodeoxycholic acid (HDCA). By using newly developed UPLC-MS/MS methods, we found vancomycin increased fecal excretion of allopregnanolone sulfate (PM4S) and PM5S during late gestation and thus maintaining the relative stability of serum PM4S and PM5S, which play an important role in BA metabolism. Further study in mice showed that pregnant mice have higher serum and liver TBA levels compared with nonpregnant mice, and PM5S administration induced higher gallbladder TBA levels and TBA pool in pregnant mice. In addition, after oral vancomycin, the continuously decreased *Parabacteroides* genus, potentially enriched with genes encoding steroids sulfatase, may explain the increased fecal PMSs excretion in pregnant sows. Taken together, our study provides the evidence that pregnancy-induced elevation of BA levels in sow is likely regulated by manipulation of gut microbiota, which offer new insights into the prevention and treatment of disrupted BA homeostasis during pregnancy by targeting specific microbiota.

## Introduction

Normal pregnancy is often associated with gradually increase in serum BA levels (Brites et al., 1998; Milona et al., 2010). Although the increase of serum BA in most women remain within normal range, a subset of women with serum BA level above reference ranges develop hypercholanemia or even intrahepatic cholestasis of pregnancy (ICP; Pascual et al., 2002; Glantz et al., 2004). The elevated maternal serum BA leads to accumulation of BA in fetal tissues, and thus increases the risk of fetal mortality and metabolic disease of offspring (Papacleovoulou et al., 2013; Zhang et al., 2015).

Evidence that disruption of BA homeostasis induce adverse fetal outcomes indicates that alleviating maternal elevated BA may improve fetal and offspring outcomes (Geenes et al., 2014). However, limited intervention strategies to remedy increased serum BA during pregnancy are available. It has long been recognized that antibiotic treatment can induce profound changes in BA metabolism (Canzi et al., 1985; Zhang et al., 2014) through microbiota mediation of BA deconjugation (Begley et al., 2006), oxidation and 7α-dehydroxylation (Ridlon et al., 2006; Lefebvre et al., 2009). Recently, our study in pregnant sows have identified a role for PMSs in dysregulation of maternal BA metabolism during pregnancy (Wang et al., 2022). UDCA administration, as a useful treatment for cholestasis, failed to reduce the production or enhance the elimination of PM4S and PM5S (Estiu et al., 2015). Notably, recent studies in pregnant women and mice have demonstrated that the alterations in the intestinal microbiome and metabolome in pregnancy contribute to hypercholanaemia during pregnancy through impairing enterohepatic feedback on BA synthesis (Ovadia et al., 2019). Thus, further study is warranted to explore the potential role of microbiota in regulating BA metabolism through PMSs processing during pregnancy.

Steroids excreted in the bile are mainly in sulfated conjugation forms. As free steroids are absorbed more rapidly than sulfated steroids, sulfation of steroids limits their reabsorption through intestine and thus stimulates their fecal excretion (Eriksson, 1971; Huijghebaert et al., 1984; Eyssen et al., 1985). However, gut bacteria, especially in the cecum and colon, possess steroid sulfatase (STS) activity, which is responsible for hydrolysis of aryl and alkyl steroid sulfates, and thus contribute to improve intestinal reabsorption of steroids after desulfation (Huijghebaert et al., 1984; Eyssen et al., 1985; Van Eldere et al., 1987, 1988; Robben et al., 1988). However, it is not clear whether antibiotics-mediated modification of microbiota can prevent the dysregulation of maternal BA. There are many similarities between pregnant humans and sows regarding the anatomy, genetics and physiology, gestation length, multiple pregnancy, BA composition and placental secretion of estrogen and progesterone (Knight and Kukoly, 1990; Barrera et al., 2007; Groenen et al., 2012), these factors suggest that results observed in human ICP have important implications for pregnant sows and fetal survival. Consistent with results in pregnant women and mice (Pascual et al., 2002; Milona et al., 2010), our previous study revealed the hypercholanemia in pregnant sows (Wang et al., 2019b). Moreover, PM5S does exist in pregnant sows and mice, and its regulation role on BA metabolism have been demonstrated in non-pregnant mice and pregnant sows (Abu-Hayyeh et al., 2013; Wang et al., 2022).Therefore, the aim of the current study was to test whether oral vancomycin, which is nonabsorbable from the intestine and shown to impact intestinal BA metabolism (Vrieze et al., 2014), could be used to modify the gut-liver metabolism of BA and progesterone and thereby prevent hypercholanemia in pregnant sows.

## Materials and methods

### Animals and samples

All experiments performed on pregnant sows and mice were approved by the Animal Care and Use Committee of Sichuan Agricultural University (Ethic Approval Code: SCAUAC201905-1). Pregnant sows were individually housed and artificially inseminated by using semen from Duroc boars. The lighting schedule was provided from 08:00 to 18:00 and ambient temperature was kept at 16–25°C. Sows were implanted with catheters at external jugular vein on day 60 of gestation (G60) as described (Takken and Williams, 1981). Considering the local use of antibiotics at the surgical site, we made sows have 1 week to restore and to adapt to experimental treatment. A total of 19 sows were assigned to control group (CON) and vancomycin group (VAN), with ten sows in CON group and nine sows in VAN group. The VAN group was administered subtherapeutic vancomycin (Amersco, Atlanta, GA, United States) with addition levels at 20 mg/kg diet according to previous study (Blake et al., 2003), vancomycin was mixed with corn starch to 10 g, and fed in the diet from G60 to the day of parturition (L0). The CON group was fed only 10 g corn starch in the diet. The feed intake from G60 to G90 was 2.45 kg/day and thereafter 3.06 kg/day till the farrowing day. Pregnant sows were transferred to farrowing rooms about 7 days prior to the predicted farrowing date and housed in individual crates. The experimental diets for each phase were formulated according to the Nutrient Requirements of Swine (National Research Council, 2012), the ingredients and composition of basal diet was shown in Supplementary Table S1. From G68 to L0, blood samples were collected *via* catheters every 7–8 days after 12-h fasting. Maternal feces were collected at G90 and G105, respectively, as constipation is common occurs around farrowing.

Male and female C57BL/6Cnc mice with 6 weeks old were purchased from Vital River Laboratory Animal Technology Co. Ltd. (Beijing, China), and breed at 10–11 weeks old. Briefly, two female mice were housed with one male mice from 20:00 till 08:00 in the next day, the successfully pregnancy of mice was determined by the detection of vaginal plugs after breeding and body weight gain within 8 days of gestation according to previous studies (Leclercq et al., 2017), otherwise the procedure would be repeated. Mice were further individually housed per cages after breeding, and free access to water. The lighting schedule was provided from 08:00 to 20:00 and ambient temperature was kept at 21–23°C. A total of 21 female C57BL/6Cnc mice were randomly assigned to nonpregnant group, pregnant group and PM5S group. According to previous study (Abu-Hayyeh et al., 2013), mice were orally gavaged with PM5S (500 mg/kg) or 0.5% carboxymethylcellulose at 17:30 and 22:30 on day 16 of gestation, 8:30 and 13:30 on day 17 of gestation, respectively. Liver, gallbladder, gut tissues of mice were weighted and stored at −80°C after euthanizing by CO_2_, serum samples were also acquired after centrifuge at 3000 × g for 10 min and then stored at −80°C.

### TBA and BA profile analysis

The TBA levels was measured by the enzymatic cycling method assay kits (Kehua Bio-Engineering Co., Ltd., Shanghai, China) according to the manufacturer’s specifications. A total of 72 species BA in serum were measured by ultrahigh performance liquid chromatography/multiple-reaction monitoring-mass spectrometry (UPLC/MRM-MS) as described (Wang et al., 2019b) in the Metabolomics Innovation Centre (TMIC). A total of 16 species BA in feces was also measured by ultraperformance liquid chromatography-mass spectrometry (UPLC-MS) as described (Yang et al., 2008).

### Fibroblast growth factor 19

Serum concentration of FGF19 were measured by porcine specific ELISA kit (RayBiotech Inc., GA, United States), the detailed methods was conducted according to the manufacturer’s specifications.

### Estradiol, progesterone and sulfated progesterone metabolites

Serum estradiol was measured by estradiol radioimmunoassay kits (Iodine[125I] Estradiol radioimmunoassay Kit, Beijing North institute of Biotechnology, Beijing, China) according to the manufacturer’s specifications. Progesterone, PM4S, and PM5S were measured by the ultra-performance liquid chromatography coupled to tandem mass spectrometry (UPLC-MS/MS) system (ACQUITY UPLC-Xevo TQ-S, Waters Corp, MA, United States) according to previous literature (Wang et al., 2022). Briefly, the Multiple Reaction Monitoring (MRM) transitions of the progesterone (315.2/97.0), PM4-S (397.1/97.2), and PM5-S (397.1/97.2) were monitored. All steroids were separated on an ACQUITY UPLC BEH C18 column (2.1 × 100 mm, 1.7 μm) using the following gradient: 50% B for 3 min, 80% for 9 min, and 100% for 0.5 min. The mobile phase B was composed of methanol with 5 mM ammonium acetate. The flow rate and injection volume were 0.4 ml/min and 5 μl, respectively.

### 16S rRNA gene sequencing of the intestinal microbiome

The feces bacteria were extracted by the commercial assay kits (Omega Bio-Tek Inc.Norvross, GA, United States). After DNA extraction, a portion of the 16S rRNA gene was amplified by using the 515F/806R primers (5′-GTGCCAGCMGCCGCGGTAA-3′ and 5′-GGACTACHVGGGTWTCTAAT-3′, respectively) which were designed to amplify the V4 region of the 16S rRNA gene. The PCR products were purified with a Qiagen Gel Extraction Kit and subjected to electrophoretic detection, and run on a 2% agarose gel. Library construction was used by TruSeq DNA PCR-Free Sample Preparation Kit (Illumina, San Diego, CA, United States). After quantitative analysis by Qubit and Q-PCR and passing the library test, high-throughput Illumina HiSeq (HiSeq 2,500, San Diego, CA, United States) sequencing platform 16S rRNA was used for sequencing.

### Data analysis

Data are expressed as means ± SE. Farrowing performance of sows, BA levels and composition, PMSs, and microbiota data were analyzed by using the SAS statistical package (V9.4, SAS Institute Inc., Cary, NC, United States). All data if normally distributed were analyzed by PROC GLM and least significant difference test, otherwise using the NPAR1WAY procedure if data were not normally distributed. GraphPad 8.0 was used to draw all the above data. *p* < 0.05 was considered statistically significant.

With respect to intestinal microbiota analysis, quality filtering on raw sequences was performed according to the QIIME (Version 1.7.0) quality-controlled process. Then the chimera sequence was removed by using UCHIM algorithm. Sequences were assigned to the same operational taxonomic units (OTUs) at a 97% identity using UCLUST in QIIME (Version 1.7.0). Representative sequence for each OUT was screened for further annotation. For each representative sequences from individual OTUs, the GreenGene Database was used based on Ribosome Database Project (RDP) Classifier (version 2.2) algorithm to annotate taxonomic information. α-diversity indices (Shannon and Chao 1) were calculated using QIIME software. Relative abundances of microbiota at different gestation day were log-transformed before analysis by using the formula: relative abundance of microbiota transformed = log10 relative abundance of microbiota.

## Results

### Effect of vancomycin on farrowing performance of sows during pregnancy

Vancomycin treatment have no significant effect on the reproductive performance of sows during late gestation. As shown in Table 1, there was no significant difference of initial body weight, farrowing body weight, litter size, liver litter size, stillbirth rate and average body weight between CON and VAN group. Consistently, the fasting serum levels of GLU, TG, TC, LDL-C and HDL-C at G90 and G105 were not significantly changed (Table 2).

**Table 1:** Effects of vancomycin supplementation on reproductive performance of sows.

Item	CON	VAN	*p*–value
Initial weight (kg)	176.58 ± 2.50	174.55 ± 2.66	0.54
Farrowing weight (kg)^1^	211.80 ± 2.97	213.23 ± 4.87	0.79
Litter size	11.50 ± 1.12	12.00 ± 1.68	0.82
Live litter size	9.75 ± 1.15	10.13 ± 1.41	0.88
Stillbirth rate (%)	15.22	15.63	1.00
Litter weight (kg)	13.43 ± 1.37	14.96 ± 2.32	0.77
Live litter weight (kg)	12.14 ± 1.35	13.80 ± 2.10	0.67
Average litter weight (kg)	1.18 ± 0.08	1.26 ± 0.09	0.51
Average weight of living litter (kg)	1.28 ± 0.06	1.35 ± 0.07	0.41

1 The body weight on parturition were weighed on the day 107 ± 2 of gestation. Data are expressed as means ± SE.

**Table 2:** Effects of vancomycin on blood biochemical indexes of sows during late gestation.

Item (mmol/l)	Time	CON	VAN	*p*-value
GLU	G90	3.70 ± 0.10	3.95 ± 0.48	0.56
	G105	4.13 ± 0.48	3.71 ± 0.16	0.47
TG	G90	0.34 ± 0.03	0.29 ± 0.03	0.26
	G105	0.34 ± 0.02	0.34 ± 0.03	0.99
TC	G90	1.59 ± 0.09	1.61 ± 0.15	0.90
	G105	1.71 ± 0.06	1.67 ± 0.15	0.78
LDL-C	G90	0.72 ± 0.05	0.74 ± 0.07	0.81
	G105	0.80 ± 0.05	0.79 ± 0.07	0.90
HDL-C	G90	0.53 ± 0.03	0.51 ± 0.06	0.75
	G105	0.58 ± 0.03	0.55 ± 0.05	0.59

GLU, glucose; TG, triglyceride; TC, total cholesterol; LDL-C, low density lipoprotein cholesterol; HDL-C, high-density lipoprotein cholesterol. Data are expressed as means ± SE.

### Effect of vancomycin on serum BA pattern during pregnancy

**Figure 1:**
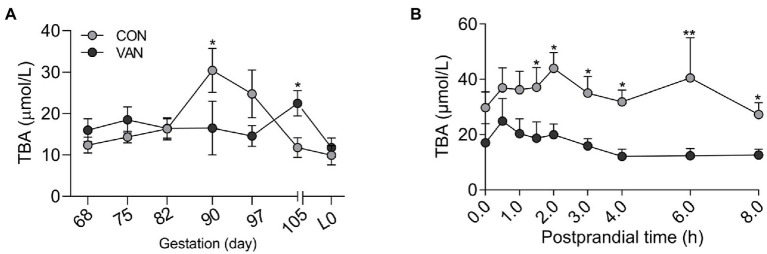
**(A)** The levels of total bile acids (TBA) in maternal peripheral serum after oral VAN from G68 till the farrowing. **(B)** Dynamic changes of maternal peripheral serum TBA within 8-h postprandial at G90 in the CON and VAN groups. Data are shown as means ± SE, ^*^p < 0.05, ^**^p < 0.01.

To test whether BA metabolism during pregnancy could be improved by regulating gut microbiota, oral vancomycin, was administrated during the second half of pregnancy. The control (CON) sows exhibited a peak serum TBA level at G90, which is significantly higher (*p* < 0.05) than at G68 (Figure 1A). In contrast, in the VAN sows, there were no differences (*p* > 0.05) in serum TBA levels among the time points and trended downward as pregnancy advanced. Moreover, the serum TBA levels at G90 was lower (*p* < 0.05) in VAN group compared with CON group, though serum TBA levels at G105 showed the opposite trend (Figure 1A). In addition, the postprandial serum TBA levels, proposed as a sensitive clinical test of liver function (LARusso et al., 1974), were significantly lower (*p* < 0.05) during the 2–8 h postprandial period in VAN than CON sows at G90, the peak levels of TBA during pregnancy (Figure 1B). These results suggest that oral VAN treatment reduced maternal TBA levels.

**Figure 2:**
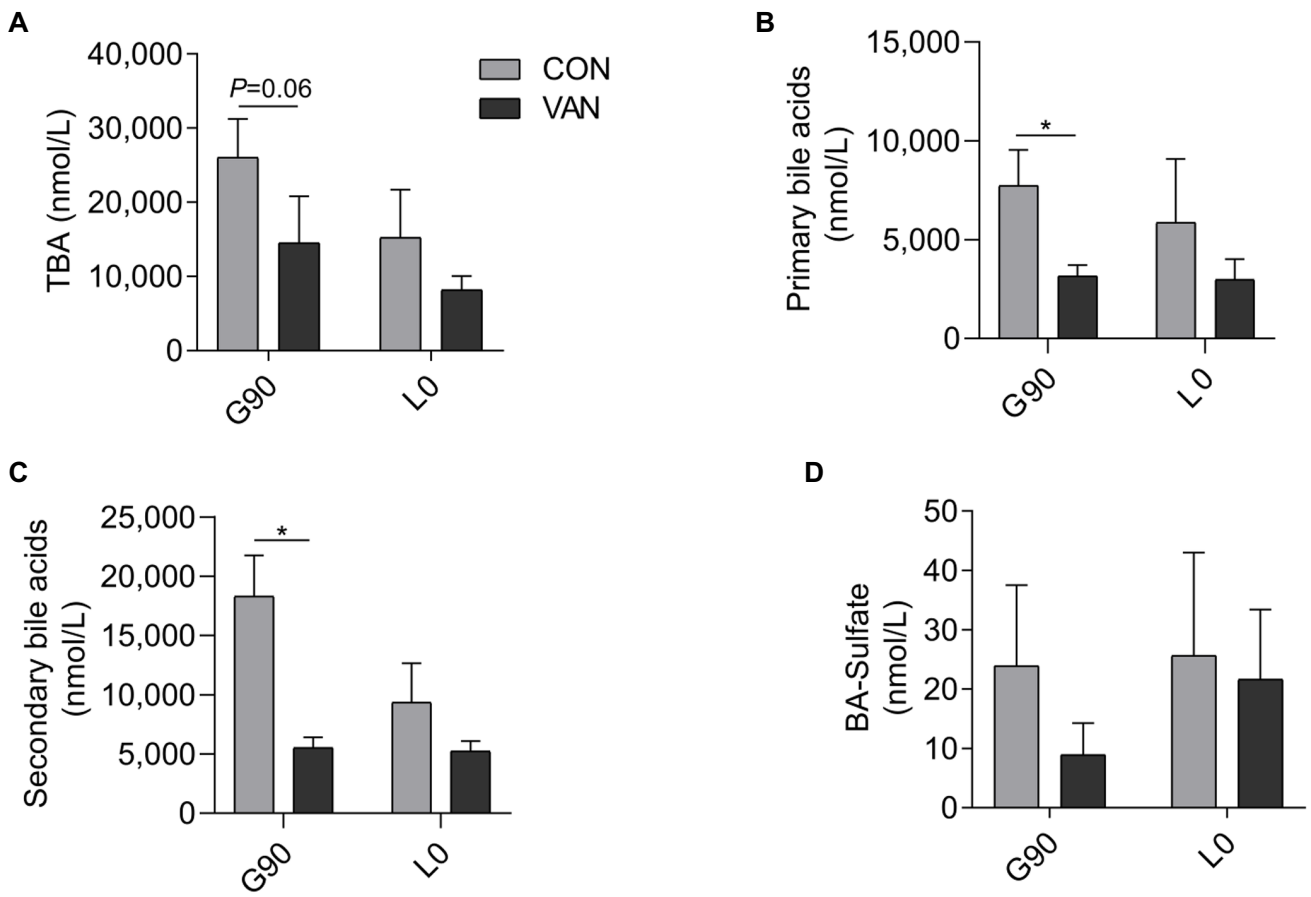
Comparison of maternal serum **(A)** TBA through calculated all individual BA through UPLC-MS/MS, **(B)** primary BA, **(C)** secondary BA, and **(D)** BA-Sulfate after VAN treatment at G90 and L0, respectively. Data are shown as means ± SE, ^*^p < 0.05.

**Figure 3:**
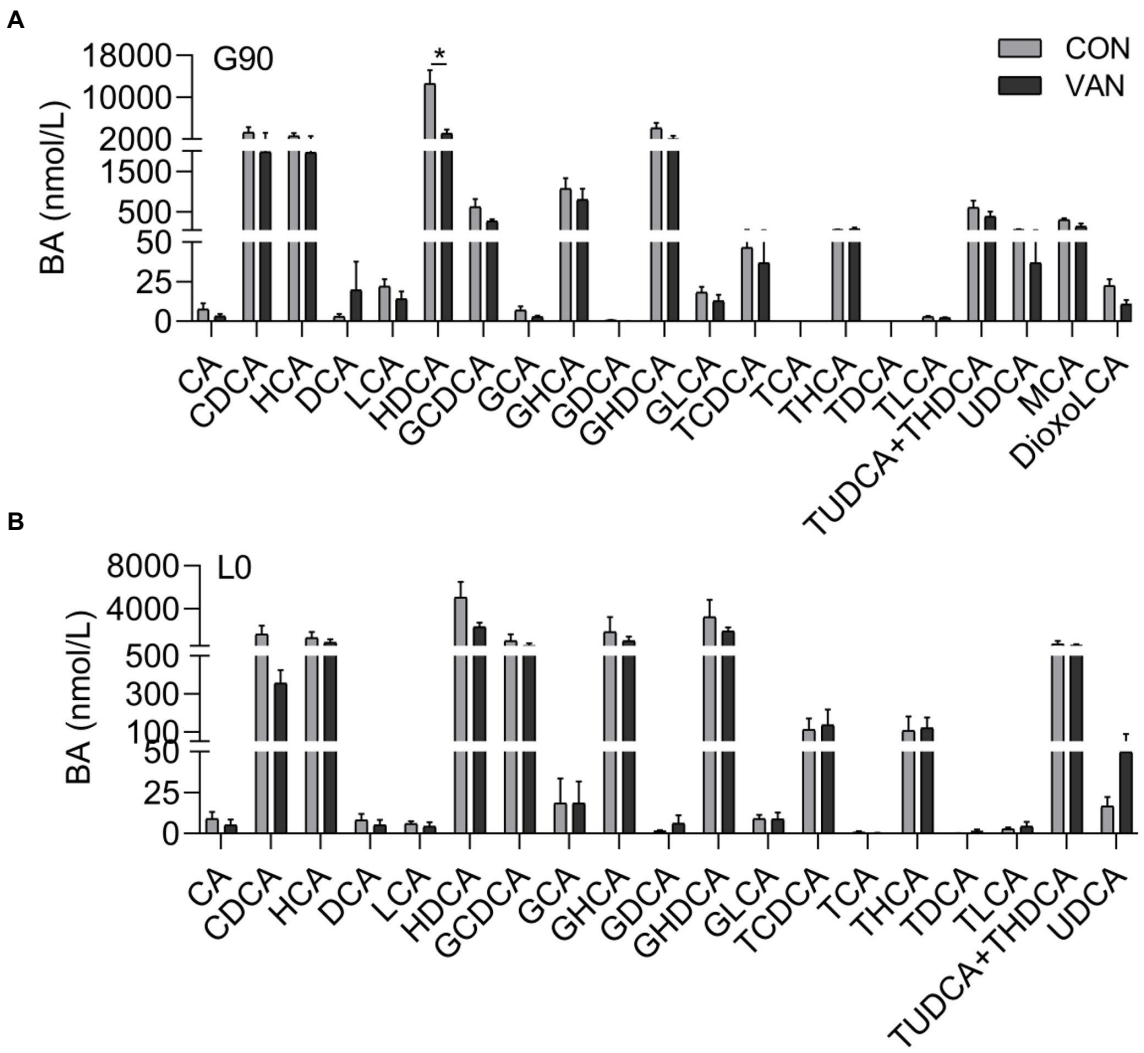
Comparison of serum BA profile between CON and VAN at **(A)** G90, and **(B)** L0, respectively. Data are shown as means ± SE, ^*^p < 0.05.

In addition to TBA, the BA profile also changed after VAN treatment. The TBA analysis based on the by calculating the sum of the 72 kinds of BA species measured by UPLC/MRM-MS were similar with TBA measured by enzymatic cycling method assay kits at G90 and L0, respectively (Figure 2A). In addition to TBA, the BA profile also changed after VAN treatment. BA profile analysis showed that lower (*p* < 0.05) TBA in VAN compared with CON at G90 was mainly attributed to lower primary BA and secondary BA, especially hyodeoxycholic acid (HDCA; Figures 2B,C, 3A,B). BA-Sulfation and BA-Glucuronidation play important roles in BA detoxification during cholestasis upon rising of BA levels (Assem et al., 2004; Trottier et al., 2006). The BA-Sulfate was not significantly (*p* > 0.05) changed after VAN treatment (Figure 2D). Moreover, BA-Glucuronide was not detectable.

### Effect of vancomycin on fecal BA during pregnancy

**Figure 4:**
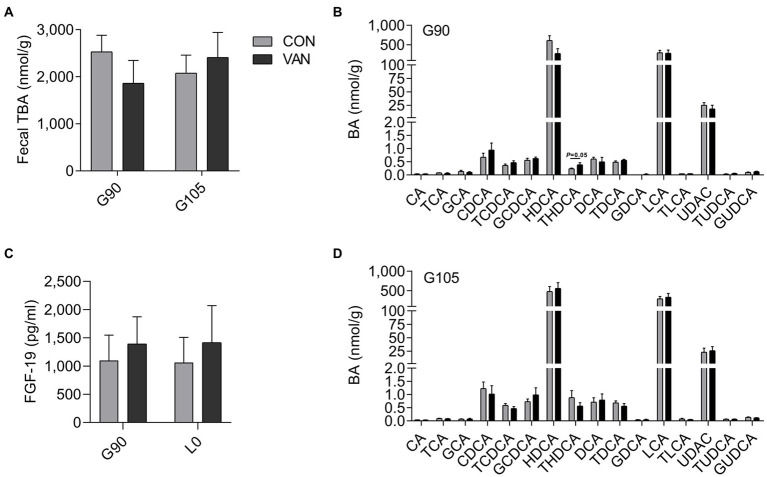
**(A,B,D)** Comparison of fecal TBA and BA profile between CON and VAN at G90 and G105, respectively. **(C)** Comparison of serum FGF19 levels between CON and VAN at G90 and L0, respectively. Data are shown as means ± SE.

To better understand the regulatory role of VAN on maternal BA, we analyzed the TBA levels and BA profile in feces. The fecal TBA concentration and BA profile were not different (*p* > 0.05) between CON and VAN group at both G90 and G105 (Figures 4A,B,D). Consistently, the serum FGF19, the intestinal FXR targeted protein, was also not significantly different (*p* > 0.05; Figure 4C).

### Effect of vancomycin on PMSs metabolism during pregnancy

**Figure 5:**
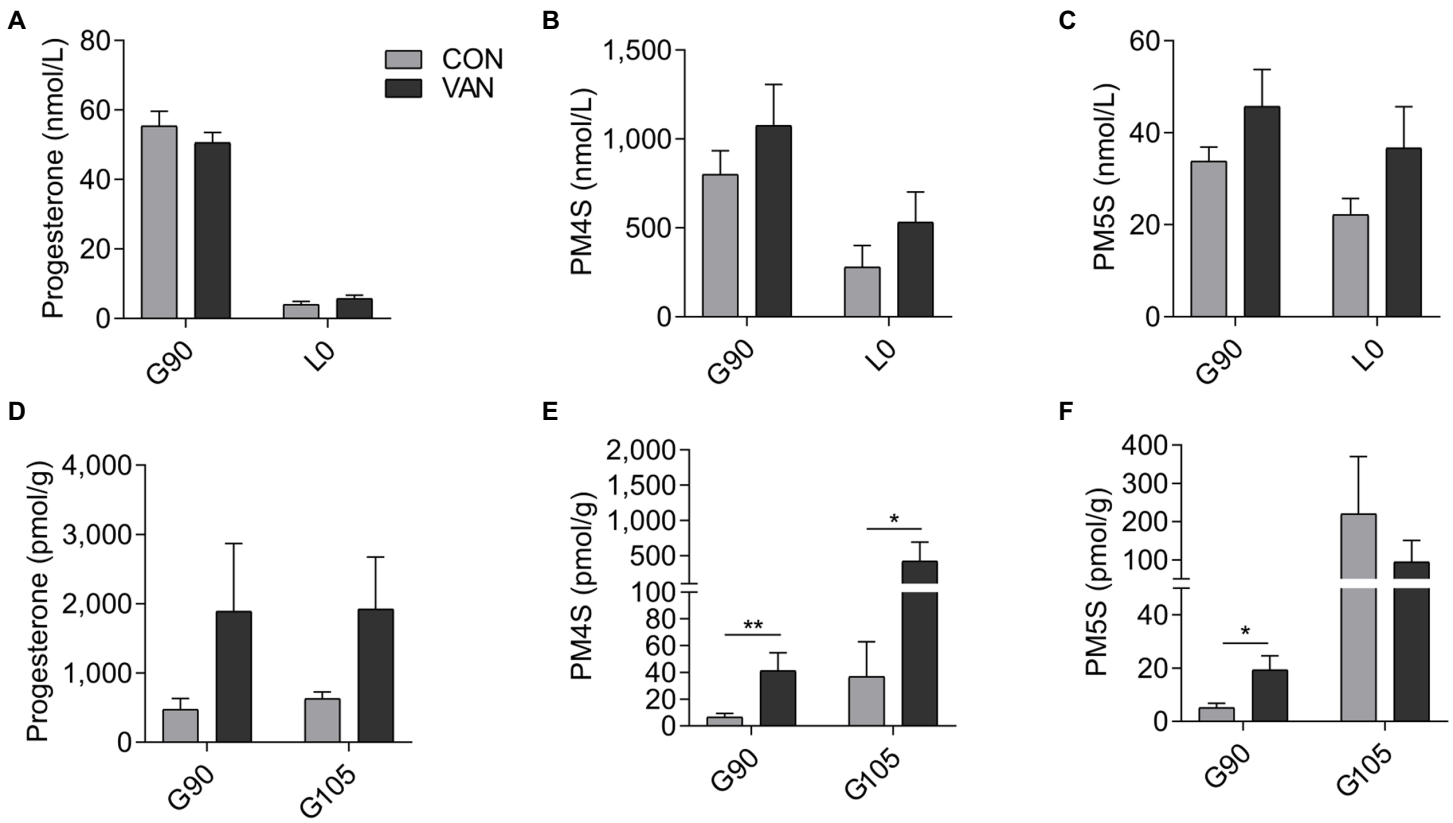
The levels of **(A)** progesterone, **(B)** PM4S, **(C)** PM5S in maternal peripheral serum, and **(D)** progesterone, **(E)** PM4S and **(F)** PM5S in feces after VAN administration at G90 and L0. Data are shown as means ± SE, ^*^p < 0.05, ^**^p < 0.01.

**Figure 6:**
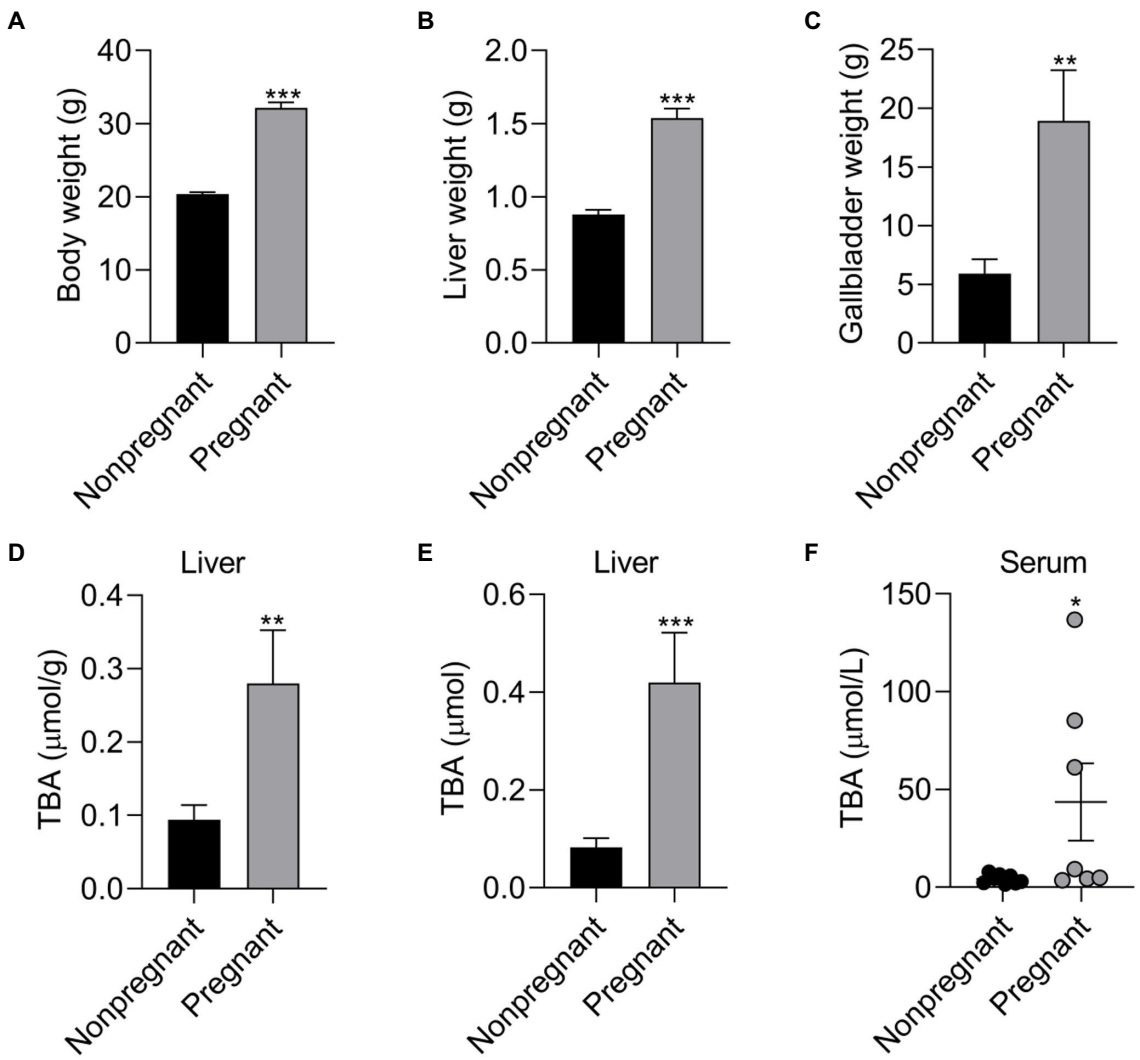
The effect of pregnancy on the body composition and BA level of female mice. The effect of pregnancy on **(A)** body weight, **(B)** liver weight and **(C)** gallbladder weight of female mice; The effect of pregnancy on **(D)** TBA concentration, **(E)** TBA levels of liver, and **(F)** serum TBA levels of female mice. Data are shown as means ± SE, ^*^p < 0.05, ^**^p < 0.01, ^***^p < 0.001.

**Figure 7:**
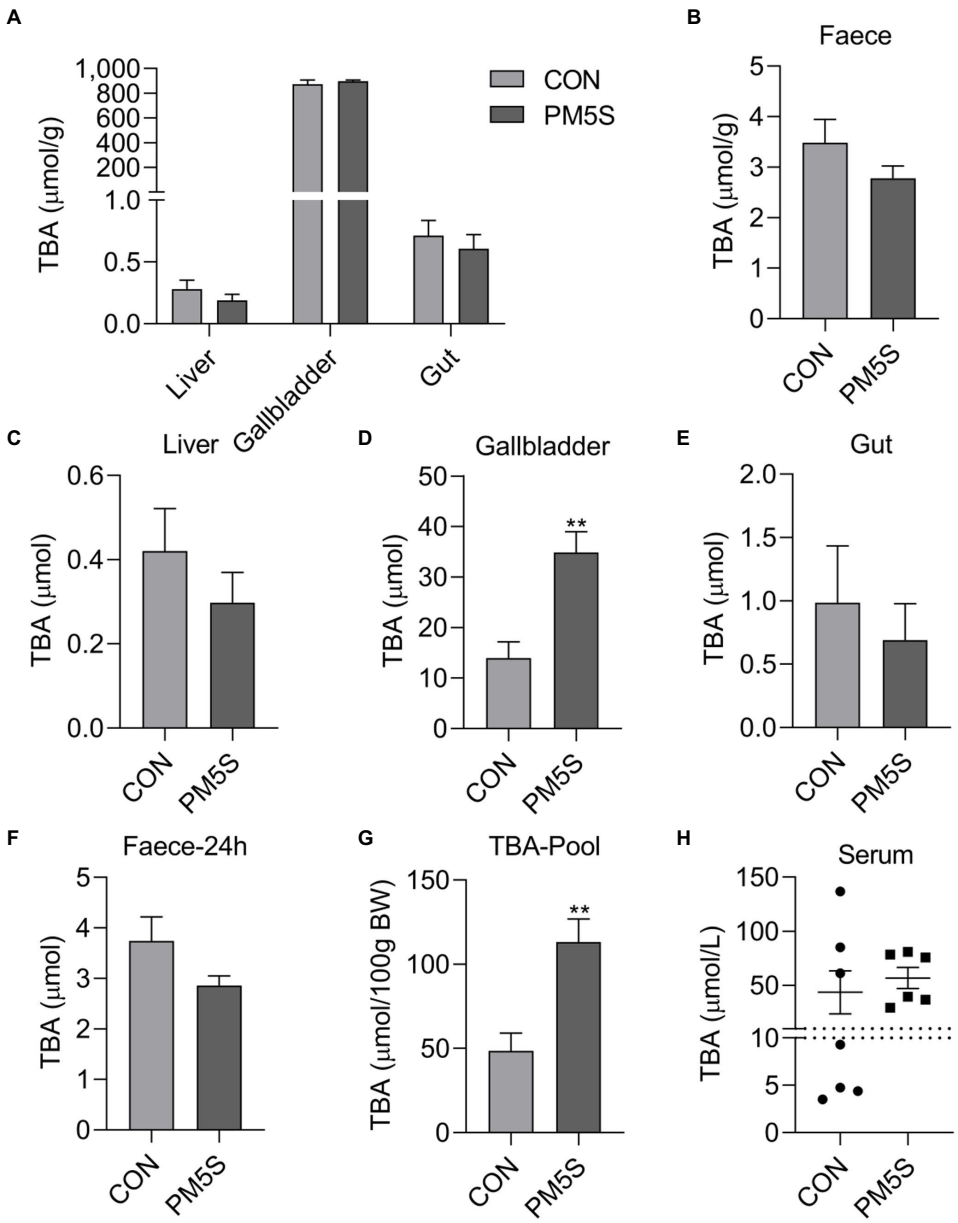
PM5S treatment changed BA metabolism of pregnant mice. The effect of PM5S treatment on TBA levels of **(A)** liver, gallbladder, gut and **(B)** feces in pregnant mice; **(E)**, The effect of PM5S treatment on TBA content of **(C)** liver, **(D)** gallbladder, **(E)** gut and **(F)** fecal BA secretion within 24-h in pregnant mice; **(G)**, The effect of PM5S treatment on **(G)** TBA pool and **(H)** serum TBA levels in pregnant mice. Data are shown as means ± SE, ^**^p < 0.01.

In the present study, VAN treatment failed to change the maternal serum levels of progesterone and estradiol (*p* > 0.05) between CON and VAN group at each time point evaluated (Figure 5A; Supplementary Figure S1). Moreover, serum levels of both PM4S and PM5S in the VAN group remained (*p* > 0.05) relatively stable from G90 to L0. Both PM4S and PM5S were not different between treatments at G90 or L0 (Figures 5B,C). Noteworthy, fecal PM4S and PM5S were higher (*p* < 0.05) in VAN than in CON group at G90, while fecal progesterone was not different between CON and VAN group at G90 and G105, respectively (Figures 5D–7F). These observations indicated increased fecal PM4S and PM5S excretion after VAN treatment.

### Effect of PM5S administration on BA metabolism in pregnant mice

To reveal the relationship between pregnancy and BA metabolism in pregnant mice and nonpregnant mice with same weeks. Pregnancy has significant effect on BA metabolism of mice. Compared with nonpregnant mice, pregnant mice have higher (*p* < 0.05) body weight, liver weight, gallbladder weight, moreover, the TBA concentration and TBA levels in liver and serum TBA levels were higher (*p* < 0.05) in pregnant mice compared with nonpregnant mice (Figure 6). To further investigate the regulation role of PMSs on BA metabolism, pregnant mice were administrated PM5S twice a day at day 16 and 17 of gestation. PM5S treatment induced higher (*p* < 0.05) TBA levels in gallbladder and TBA pool, though the TBA levels in serum, liver, gallbladder, gut and feces within 24 h were not different (*p* < 0.05; Figure 7). These results demonstrated the regulation role of PM5S on BA metabolism.

### Vancomycin administration decreased the relative abundance of microbiota involved in desulfation

To test whether the change of sulfated progesterone metabolism following oral VAN was associated with intestinal microbiota, we determined the microbiota profile of fecal samples collected at G90 and G105, respectively, by using 16S rRNA gene sequencing. A total of 3,243,120 effective sequences were obtained in CON and VAN groups, with an average of 72,069 ± 1,642 effective sequences in each sample. A total of 60,448 OTUs were also obtained in the two groups, with an average of 1,343 ± 12 OTUs per sample. The Rarefaction Curve of fecal samples in CON and VAN group at both G90 and G105 indicated the sequencing depth could cover almost all bacterial flora (Supplementary Figure S2). The Chao 1 index was higher in VAN group compared with CON group at G90, though the Chao 1 was not changed at G105 and Shannon index was not different at both G90 and G105 between CON and VAN group (Table 3).

**Table 3:** Comparison of alpha metrics’ difference in control group (CON) and vancomycin group (VAN).

Item	Time	CON	VAN	*p*-value
Species	G90	1152.60 ± 22.29	1195.00 ± 16.73	0.40
	G105	1118.40 ± 16.78	1139.40 ± 6.19	0.28
Shannon	G90	7.75 ± 0.16	7.86 ± 0.15	0.62
	G105	7.62 ± 0.12	7.68 ± 0.09	0.70
Chao 1	G90	1325.82 ± 26.34	1423.81 ± 33.56	0.03
	G105	1295.75 ± 19.88	1340.01 ± 27.39	0.20

Means in rows with different superscript litters are significantly different (*p* < 0.05). Data are expressed as means ± SE.

Indeed, oral VAN induced changes of gut microbiota composition. At phylum level, VAN significantly increased (*p* < 0.05) *Verrucomicrobia* and *Planctomycetes* at G90 and *Fibrobacteres*, *Planctomycetes*, *Cyanobacteria* at G105 (Table 4). At genus level, *Streptococcus* and *Escherichia* were significantly decreased (*p* < 0.05) while *Succinivibrio*, *Ruminococcus* and *Fibrobacter* were significantly increased (*p* < 0.05) after VAN treatment at G105. Particularly, we observed the consistently lower *Parabacteroides* (1.81% vs. 0.89%, *p <* 0.05; 1.29% vs. 0.81%, *p* < 0.05), consistently higher *Desulfovibrio* (0.16% vs. 0.25%, *p* < 0.01; 0.12% vs. 0.23%, *p* < 0.01) and *Dore*a (0.16% vs. 0.27%, *p* < 0.05; 0.15% vs. 0.29%, *p* < 0.01) after VAN treatment at both G90 and G105 (Tables 5, 6).

**Table 4:** The relative abundance of microflora (top 10) at phylum level between CON and VAN group (%).

	CON	VAN	Pooled SEM	*p*-value
Day 60 of gestation				
Firmicutes	47.17	46.71	4.42	0.93
Bacteroidetes	35.29	40.14	5.22	0.34
Spirochaetes	7.02	4.22	1.68	0.07
Proteobacteria	3.80	2.89	1.55	0.55
Verrucomicrobia	1.59	1.61	0.41	0.86
Euryarchaeota	0.92	0.27	0.80	0.28
Tenericutes	2.29	2.39	0.39	0.81
Fibrobacteres	0.52	0.64	0.29	0.91
Planctomycetes	0.11	0.04	0.04	0.14
Cyanobacteria	0.16	0.15	0.04	0.58
Day 90 of gestation				
Firmicutes	45.51	45.99	3.84	0.96
Bacteroidetes	38.71	38.21	3.93	0.77
Spirochaetes	6.87	5.01	1.90	0.74
Proteobacteria	3.26	3.27	1.12	0.82
Verrucomicrobia	1.23	2.11	0.34	0.049
Euryarchaeota	0.12	0.20	0.06	0.08
Tenericutes	2.40	2.95	0.68	0.48
Fibrobacteres	0.71	0.66	0.40	0.56
Planctomycetes	0.03	0.13	0.03	0.001
Cyanobacteria	0.12	0.21	0.04	0.12
Day 105 gestation				
Firmicutes	45.11	40.77	3.35	0.21
Bacteroidetes	41.44	39.51	3.45	0.72
Spirochaetes	4.32	7.46	1.62	0.07
Proteobacteria	3.52	5.23	1.43	0.13
Verrucomicrobia	1.59	2.88	0.83	0.09
Euryarchaeota	0.39	0.32	0.18	0.66
Tenericutes	2.20	1.73	0.55	0.61
Fibrobacteres	0.22	0.50	0.12	0.03
Planctomycetes	0.02	0.12	0.04	0.005
Cyanobacteria	0.08	0.20	0.03	0.004

**Table 5:** The relative abundance of microflora (top 35) at genera level between CON and VAN group at day 90 of gestation (%).

	CON	VAN	*p*-value
*Prevotella*	6.82 ± 1.20	5.53 ± 1.30	0.46
*Treponema*	6.59 ± 1.25	4.74 ± 0.63	0.34
*Succinivibrio*	0.96 ± 0.26	1.08 ± 0.28	0.62
*Streptococcus*	2.11 ± 0.74	0.83 ± 0.11	0.18
*Escherichia*	1.01 ± 0.73	0.32 ± 0.04	0.85
*Parabacteroides*	1.81 ± 0.49	0.89 ± 0.05	0.04
CF231(Paraprevotellaceae)	2.19 ± 0.19	2.96 ± 0.75	0.21
Methanobrevibacter	0.08 ± 0.03	0.14 ± 0.05	0.30
[*Prevotella*]	1.49 ± 0.14	1.22 ± 0.20	0.29
*Anaerovibrio*	0.41 ± 0.05	0.52 ± 0.12	0.34
*Ruminococcus*	1.22 ± 0.18	1.33 ± 0.12	0.54
*Oscillospira*	2.20 ± 0.18	2.62 ± 0.08	0.15
YRC22	1.22 ± 0.26	0.72 ± 0.15	0.22
Fibrobacter	0.71 ± 0.26	0.66 ± 0.21	0.46
Paludibacter	0.52 ± 0.07	0.56 ± 0.09	0.75
Phascolarctobacterium	0.57 ± 0.13	0.84 ± 0.05	0.07
p^−75^-a5	0.81 ± 0.22	0.55 ± 0.08	0.71
*Bacteroides*	1.12 ± 0.12	0.93 ± 0.05	0.43
*Lactobacillus*	0.51 ± 0.11	0.46 ± 0.05	0.71
*Mitsuokella*	0.18 ± 0.05	0.14 ± 0.03	0.56
*Coprococcus*	0.58 ± 0.05	0.51 ± 0.06	0.38
*Epulopiscium*	0.23 ± 0.03	0.21 ± 0.03	0.67
02d06	0.79 ± 0.10	1.05 ± 0.17	0.19
*Roseburia*	0.34 ± 0.02	0.56 ± 0.16	0.09
*Faecalibacterium*	0.23 ± 0.09	0.15 ± 0.02	0.71
*Campylobacter*	0.27 ± 0.07	0.21 ± 0.05	0.55
*Megasphaera*	0.09 ± 0.02	0.10 ± 0.02	0.46
vadinCA11	0.04 ± 0.02	0.06 ± 0.03	0.33
RFN20	0.18 ± 0.02	0.22 ± 0.05	0.39
*Dorea*	0.16 ± 0.02	0.27 ± 0.03	0.02
*Clostridium*	0.22 ± 0.02	0.24 ± 0.02	0.43
*Lachnospira*	0.14 ± 0.02	0.12 ± 0.01	0.30
*Sutterella*	0.12 ± 0.03	0.10 ± 0.04	0.36
SMB53	0.22 ± 0.02	0.25 ± 0.03	0.45
*Desulfovibrio*	0.16 ± 0.02	0.25 ± 0.02	0.004

**Table 6:** The relative abundance of microflora (top 35) at genera level between CON and VAN group at day 105 of gestation (%).

	CON	VAN	*P*-value
*Prevotella*	7.25 ± 1.39	6.00 ± 0.74	0.90
*Treponema*	4.08 ± 0.84	6.96 ± 1.44	0.09
*Succinivibrio*	0.69 ± 0.14	2.25 ± 0.78	0.01
*Streptococcus*	2.50 ± 0.84	0.61 ± 0.12	0.03
*Escherichia*	1.58 ± 0.87	0.18 ± 0.02	0.01
*Parabacteroides*	1.29 ± 0.13	0.81 ± 0.10	0.03
CF23 (*Paraprevotellaceae*)	2.97 ± 0.40	2.75 ± 0.31	0.90
*Methanobrevibacter*	0.16 ± 0.06	0.16 ± 0.06	1.00
*[Prevotella]*	1.59 ± 0.24	1.56 ± 0.52	0.81
*Anaerovibrio*	0.69 ± 0.22	0.61 ± 0.08	0.39
*Ruminococcus*	1.01 ± 0.07	1.36 ± 0.14	0.02
*Oscillospira*	2.50 ± 0.19	2.48 ± 0.16	0.95
*YRC22*	0.69 ± 0.16	0.49 ± 0.11	0.46
*Fibrobacter*	0.22 ± 0.03	0.50 ± 0.17	0.04
*Paludibacter*	0.45 ± 0.10	0.48 ± 0.17	0.90
*Phascolarctobacterium*	0.90 ± 0.25	0.72 ± 0.12	0.71
p^−75^-a5	0.67 ± 0.09	0.35 ± 0.08	0.04
*Bacteroides*	0.92 ± 0.09	0.75 ± 0.15	0.11
*Lactobacillus*	0.68 ± 0.14	0.54 ± 0.07	0.81
*Mitsuokella*	0.18 ± 0.04	0.42 ± 0.26	0.46
*Coprococcus*	0.72 ± 0.12	0.53 ± 0.04	0.18
*Epulopiscium*	0.33 ± 0.15	0.12 ± 0.03	0.22
02d06	0.69 ± 0.07	0.77 ± 0.05	0.47
*Roseburia*	0.34 ± 0.04	0.41 ± 0.07	0.32
*Faecalibacterium*	0.21 ± 0.04	0.22 ± 0.09	0.54
*Campylobacter*	0.19 ± 0.08	0.26 ± 0.10	0.18
*Megasphaera*	0.12 ± 0.02	0.25 ± 0.15	0.85
vadinCA11	0.22 ± 0.09	0.16 ± 0.11	0.71
RFN20	0.21 ± 0.03	0.18 ± 0.02	0.53
*Dorea*	0.15 ± 0.02	0.29 ± 0.05	0.007
*Clostridium*	0.22 ± 0.02	0.18 ± 0.02	0.32
*Lachnospira*	0.15 ± 0.03	0.11 ± 0.01	0.15
*Sutterella*	0.11 ± 0.02	0.13 ± 0.04	0.57
SMB53	0.20 ± 0.02	0.20 ± 0.02	0.85
*Desulfovibrio*	0.12 ± 0.01	0.23 ± 0.04	0.003

## Discussion

Studies in pregnant women and rodents have revealed physiological change of BA and reproductive hormones during pregnancy, particularly the role of estrogen or its glucuronidated forms (Huang et al., 2000; Milona et al., 2010; Song et al., 2014). However, glucuronidation was a minor pathway for steroid metabolism in both human, rodent, minipigs and pregnant sows (Thakare et al., 2018; Wang et al., 2019a,b). The first important finding in this study is that oral VAN administration appeared to prevent the increase in TBA during pregnancy possibly through regulating PMSs, especially PM4S and PM5S. In general, VAN treatment reduced the serum TBA at mid-pregnancy and particularly in the postprandial phase of feeding. This suppression of serum TBA was dominated by a decrease in primary and secondary BA. It has been well documented that primary BA are mainly synthesized in hepatocytes while secondary BA are mainly derived from gut microbial metabolism. Given the promoting role of PM4S and PM5S on maternal BA metabolism during pregnancy (Abu-Hayyeh et al., 2013; Estiu et al., 2015), the increased fecal excretion of PM4S and PM5S following VAN treatment may in part explain the suppression of primary BA. In support of this, our studies also demonstrated the promoting role of PM5S on BA pool. Moreover, our previous studies have shown that both PM4S and PM5S promoted BA synthesis in pig primary hepatocytes through inhibiting farnesoid X receptor (Wang et al., 2022). Consequently, the decrease of secondary BA after VAN treatment may be the carry-over effect of primary BA.

Previous studies have revealed the regulatory effect of antibiotics *via* intestinal microbiota on fecal sulfated steroids, which was the main excretion pathway of sulfated steroids (Eriksson, 1971). Likewise, we observed increased fecal PM4S and PM5S excretion following oral VAN administration. Moreover, the fecal progesterone metabolites in cholestasis patients were decreased during pregnancy (Adlercreutz and Martin, 1980). Thus, it appears promising to prevent PMSs accumulation *via* gut microbiota modification to increase their excretion from feces. However, it is especially challenging to identify which bacteria participate in the desulfation of steroids. As *desulfovibrio*, which belong to sulphate-reducing microorganisms, use sulphate as the terminal electron acceptor producing hydrogen sulphide and carbon dioxide (Zhou et al., 2011), it seems not possible to directly desulfate sulfated progesterone metabolites.Moreover, consistent with the results found in human (Vrieze et al., 2014), the consistently increased *desulfovibrio* (Gram negative bacteria) may be a compensatory result of the VAN-mediated suppression of Gram positive bacteria.Earlier studies have shown four strains of phenotypically similar *Bacteroides* species express STS activity specific for aryl steroids like estrogen sulfates (Van Eldere et al., 1988). Recently, *Parabacteroides* bacteria was identified to not only encode ≥19 sulfatases, but also possess an anaerobic sulfatase-maturating enzyme (anSME) coding gene, thus having a putative activity in desulfation of steroids (Benjdia et al., 2011). In present study, the gradual decrease in fecal *Parabacteroides* genus, suggesting reduced desulfationactivity of microbiota, may account for the increased fecal PMSs after VAN administration. The detailed effect of *Parabacteroides* on PMSs metabolism deserves further investigation.

Taken together, the findings of this study demonstrate that oral vancomycin induced change of abundance of gut microbiota, potentially enriched with genes encodingsteroids sulfatase, improved maternal BA homeostasis during pregnancy through increasing fecal PM4S and PM5S secretion. This study provides novel insights into the prevention and treatment of hypercholanaemia by targeting specific microbiota.

## Data availability statement

The data presented in the article are publicly available. The 16srRNA data have uploaded in SRA, https://www.ncbi.nlm.nih.gov/sra, with accession number PRJNA699767.

## Ethics statement

The animal study was reviewed and approved by Animal Care and Use Committee of Sichuan Agricultural University. Written informed consent was obtained from the owners for the participation of their animals in this study.

## Author contributions

PW performed and analyzed the experiments and drafted the manuscript. QC, PY, SL, HC, RL, XZ, and JL performed experiments and edited the manuscript. YZ, LC, BF, YL, and SX performed the analysis of the BA data. DW supervised the experiment and edited the manuscript. ZF conceptualized, designed, performed and analyzed the experiments and wrote the manuscript. All authors contributed to the article and approved the submitted version.

## Funding

This work was supported by the National Natural Science Foundation of China (31972603), Henan Provincial Science and Technology Research Project (grant no. 212102110161), and Doctoral Scientific Research Start-up Foundation from Henan University of Technology (grant no. 31401373).

## Conflict of interest

The authors declare that the research was conducted in the absence of any commercial or financial relationships that could be construed as a potential conflict of interest.

## Publisher’s note

All claims expressed in this article are solely those of the authors and do not necessarily represent those of their affiliated organizations, or those of the publisher, the editors and the reviewers. Any product that may be evaluated in this article, or claim that may be made by its manufacturer, is not guaranteed or endorsed by the publisher.
