# Supplementary material for: Gut microbiota involved in desulfation of sulfated progesterone metabolites: A potential regulation pathway of maternal bile acid homeostasis during pregnancy
Source: Front Microbiol. 2022 Oct 20;13:1023623. doi: 10.3389/fmicb.2022.1023623 (PMC9631449; doi:10.3389/fmicb.2022.1023623)
Supplement: Supplementary file 1 [file Data_Sheet_1.docx]

Supplementary Material


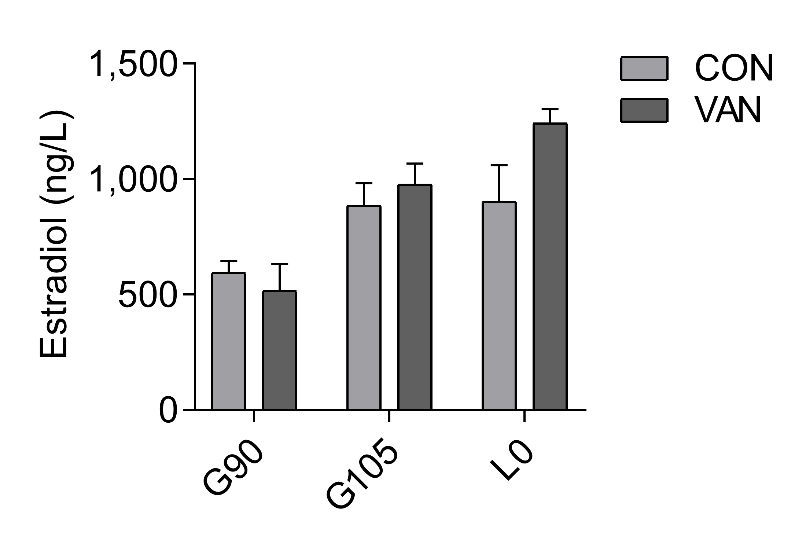


*Supplementary Figure S1. Effect of vancomycin on serum estradiol levels in pregnant sows during late gestation. Data are shown as means ± SE.*


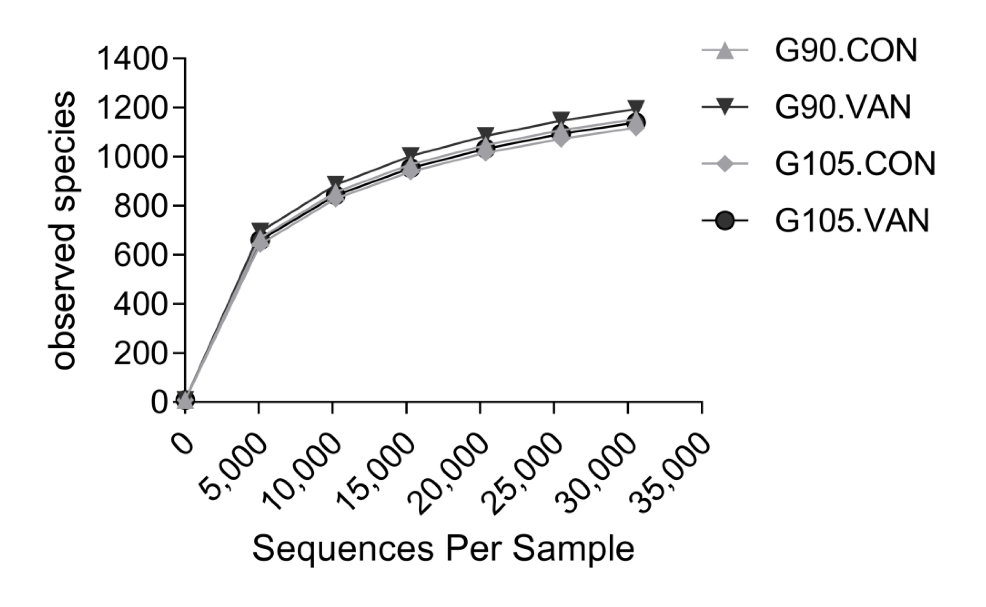


*Supplementary Figure S2. Rarefaction curves of fecal samples in CON and VAN group at G90 and G105.*

Supplemental Table S1. Ingredients and composition of basal diet

| Ingredients | % | Nutritional level | Content |
| --- | --- | --- | --- |
| Corn | 63.19 | Digestible energy, Mcal/kg | 3.08 |
| Soybean meal | 14.4 | Metabolizable energy, Mcal/kg | 2.94 |
| Wheat bran | 18.00 | Total Lysine, % | 0.70 |
| Fish meal, 67% | 1.00 | SID-Lysine, % | 0.58 |
| Limestone | 1.01 | Crude protein, % | 14.6 |
| Dicalcium phosphate | 1.21 | Calcium, % | 0.80 |
| *_L_*-Lysine-HCL, 98.5% | 0.02 | Total phosphorus, % | 0.68 |
| *_L_*-Methionine, 99% | 0.02 | Available Phosphorus, % | 0.44 |
| _L-_Threonine, 98.5% | 0.05 |  |  |
| Sodium chloride | 0.45 |  |  |
| Vitamin and minerals premix^#^ | 0.50 |  |  |
| Choline Chloride, 50% | 0.15 |  |  |
| Total | 100 |  |  |

^#^ Diet per kilogram: 165 mg Fe, 16 mg Cu, 165 mg Zn, 30 mg Mn, 0.3 mg Se, 0.3 mg I, 0.2mg Cr, 10,000 IU VA, 2,500 IU VD_3_, 44 IU VE, 2.5 mg VK, 1 mg VB_1_, 8 mg VB_2_, 3 mg VB_6_, 0.015 mg VB_12_, 17.5 mg nicotinic acid, 12.5 mg pantothenic acid, 1.32 mg folic acid and 0.2mg biotin.
